# Does Severity of Alzheimer's Disease Contribute to Its Responsiveness to Modifying Gut Microbiota? A Double Blind Clinical Trial

**DOI:** 10.3389/fneur.2018.00662

**Published:** 2018-08-15

**Authors:** Azadeh Agahi, Gholam Ali Hamidi, Reza Daneshvar, Mostafa Hamdieh, Masoud Soheili, Azam Alinaghipour, Seyyed Mohammad Esmaeili Taba, Mahmoud Salami

**Affiliations:** ^1^Physiology Research Center, Kashan University of Medical Sciences, Kashan, Iran; ^2^Department of Neurology, School of Medicine, Kashan University of Medical Sciences, Kashan, Iran; ^3^Department of Psychology, School of Medicine, Shaheed Beheshti University of Medical Sciences, Tehran, Iran; ^4^Taleghani Branch, Department of Education, Farhangian University, Qom, Iran

**Keywords:** probiotics, Alzheimer's disease, cognition, inflammation, oxidative stress, microbiota

## Abstract

Alzheimer's disease (AD) is associated with cognitive dysfunction. Evidence indicates that gut microbiota is altered in the AD and, hence, modifying the gut flora may affect the disease. In the previous clinical research we evaluated the effect of a probiotic combination on the cognitive abilities of AD patients. Since, in addition to pathological disorders, the AD is associated with changes in oxidant/antioxidant and inflammatory/anti-inflammatory biomarkers, the present work was designed to evaluate responsiveness of the inflammatory and oxidative biomarkers to the probiotic treatment. The control (CON) and probiotic (PRO) AD patients were treated for 12 weeks by the placebo and probiotic supplementation, respectively. The patients were cognitively assessed by Test Your Memory (TYM = 50 scores). Also serum concentrations of nitric oxide (NO), glutathione (GSH), total antioxidant capacity (TAC), malondialdehyde (MDA), 8-hydroxy-2′ -deoxyguanosine (8-OHdG) and cytokines (TNF-a, IL-6, and IL-10) were measured. The cognitive test and the serum biomarkers were assessed pre- and post-treatment. According to TYM test 83.5% of the patients showed severe AD. The CON (12.86% ± 8.33) and PRO (−9.35% ± 16.83) groups not differently scored the cognitive test. Not pronounced change percent was found in the serum level of TNF-α (1.67% ± 1.33 vs. −0.15% ± 0.27), IL-6 (0.35% ± 0.17 vs. 2.18% ± 0.15), IL-10 (0.05% ± 0.10 vs. −0.70% ± 0.73), TAC (0.07% ± 0.07 and −0.06% ± 0.03), GSH (0.08% ± 0.05 and 0.04% ± 0.03) NO (0.11% ± 0.06 and 0.05% ± 0.09), MDA (−0.11% ± 0.03 and −0.17% ± 0.03), 8-OHdG (43.25% ± 3.01 and 42.70% ± 3.27) in the CON and PRO groups, respectively. We concluded that the cognitive and biochemical indications in the patients with severe AD are insensitive to the probiotic supplementation. Therefore, in addition to formulation and dosage of probiotic bacteria, severity of disease and time of administration deeply affects results of treatment.

## Introduction

Alzheimer's disease (AD) is a common neurodegenerative disease accounting for 60–80% of all dementia (1). It is characterized by progressive declines in cognitive and functional abilities, neuropsychiatric symptoms, caregiver burden and premature death (2). Particularly, it is accompanied with early impairment of episodic memory (3, 4) followed by progressive deficits in short term memory leading to a final procedural memory deficit (5). AD is etiologically resulted of the extracellular accumulation of amyloid-β plaques, formation of neurofibrillary tangles, neuroinflammation, neuronal injury, and synapse loss (6). In recent studies, neuroinflammation and oxidative stress are particularly considered for their important role in the pathogenesis of AD (7, 8). Especially, growing evidences indicate that both oxidative stress and inflammation are involved in the mechanisms associated with Aβ-induced neurotoxicity (9).

Cholinesterase inhibitors such as donepezil, rivastigmin, and galantamin are approved for treatment of mild to moderate degree of AD. Also the partial NMDA receptor antagonist memantin is prescribed in the moderate to severe of AD (10). However, in spite of fanciful advances in therapeutic aims, AD still lack effective medications. Therefore, novel and effective treatments research have focused on other resources of treatment (11). The microbiota, the ecological community of commensal, symbiotic, and pathogenic microorganisms literally sharing our body space, includes more than 10 times the number of host cells to human cells (12). Emerging studies indicate that the microbiota may contribute to the regulation of multiple neurochemical and neurometabolic pathways through a complex series of highly interactive and symbiotic host-microbiome signaling systems (13–15). This interconnection of the gastrointestinal tract and the central nervous system (CNS) is known as microbiota-gut-brain axis (16) that regulates brain function and behavior. Emerging data demonstrates that certain pathologies, related to an altered microbiome, are linked to mood, stress, behavior, and cognition (17). Some complications such as cognitive disorders, oxidative stress, neuroinflammation, which are also observable in AD, are identified to be influenced by the gut flora as well as probiotics. Probiotics are defined as the living microorganisms with the beneficial effects for humans and animals when administered in a sufficient number. The most commonly used probiotics are the strains of lactic acid bacteria such as *Lactobacillus* and *Bifidobacterium*. Preclinical research shows that probiotics may improve cognitive performances in the animal models with impaired cognition (18). It is reported that probiotics inhibit oxidative stress via reducing inflammation and increasing antioxidant enzymes such as superoxide dismutase and glutathione peroxidase (19).

Test Your Memory (TYM), introduced by Brown et al. (20), is a brief test designed to detect AD. It includes a series of 10 cognitive tasks consists of orientation, ability to copy a sentence, semantic knowledge, calculation, verbal fluency, similarities, naming, visuospatial abilities, ability to do the test and recall of a copied sentence giving a possible total of 50 scores. Importantly, the developers of the test believe that education and social class have only mild effects on the TYM score (20).

In our previous work we reported for the first time the effect of a probiotic supplementation on the cognitive as well as metabolic status of the patients with AD (21). In the present work using a different formulation of probiotic combination we applied the TYM test as a cognitive tool to assess the cognitive status of the people suffering from AD treated by a mixture of probiotic bacteria. Further, because of deep changes in balance of the oxidant/antioxidant and inflammatory/anti-inflammatory biomarkers in the AD, in the present work we also considered how the serum concentration of the inflammatory and oxidative biomarkers responds to the oral bacteriotherapy.

## Materials and methods

### Participants

**Figure 1:**
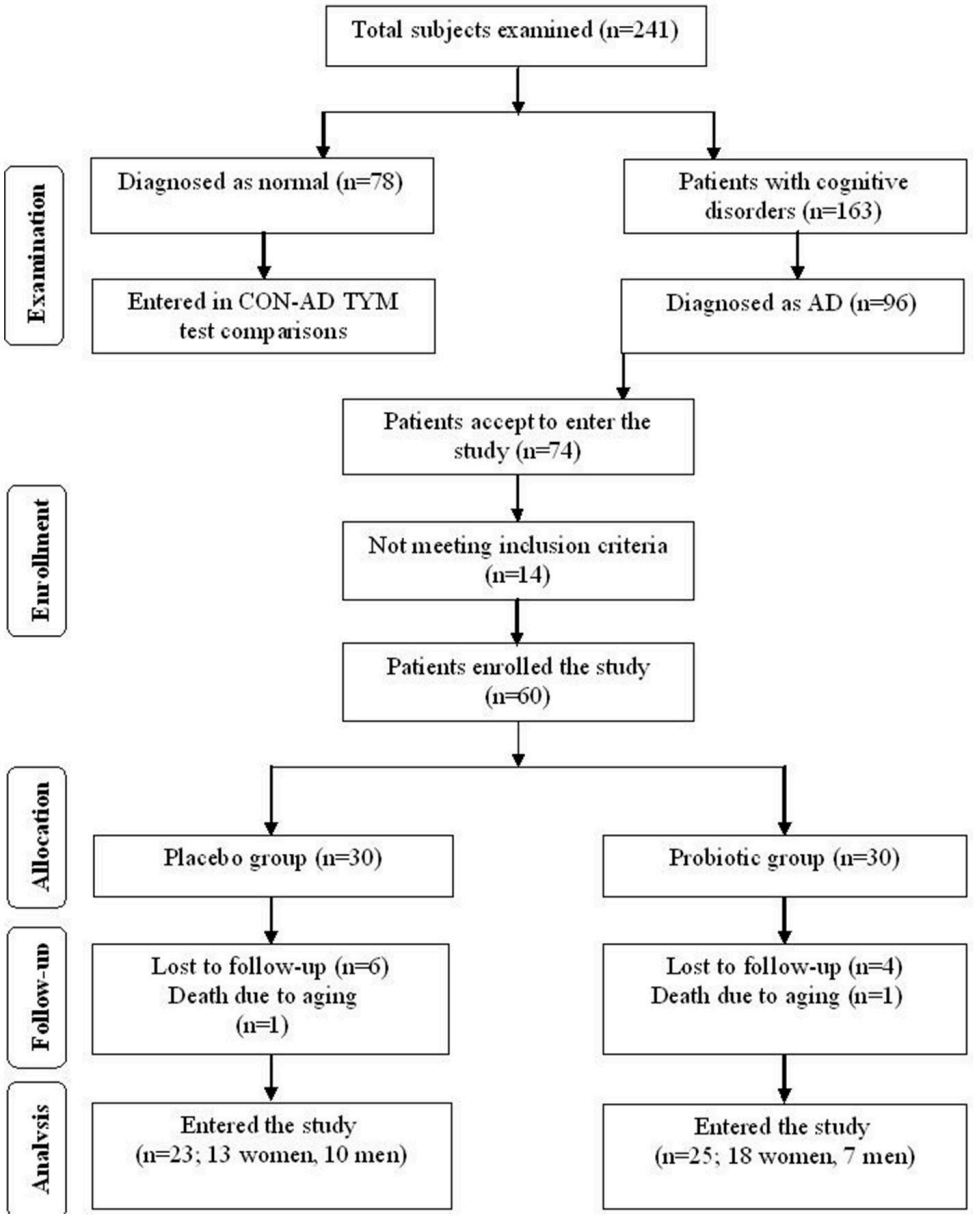
Summary of patient flow.

This clinical study was performed as a randomized, double-blind, and placebo-controlled clinical trial. Participants included in this study were people with AD (65–90 years old) residing at Emam Ali (Tehran, Iran), Golabchi (Kashan, Iran), Miad (Kashan, Ravand, Iran), and Barekat (Aran and Bidgol, Iran) Welfare Organizations between June 2017 and August 2017. AD patients were diagnosed based on the NINDS-ADRDA criteria (22) and revised criteria from the National Institute on Aging-Alzheimer's Association (23). For further proof the AD patients were compared with the cognitively intact people based on the TYM cognitive test. Accordingly, the participants gaining TYM scores in level of AD (<45 out of 50 scores) were entered the study (see section “Results” for details). Patients with metabolic disorders, chronic infections and/or other clinically relevant disorders were excluded from the study. Standard formula for clinical trials was used to calculate sample size for the study. Based on a previous study (24), considering type one error (α) of 0.05 and type two error (β) of 0.20 (power = 80%) we used 1.3 as SD and 1.1 as the difference in mean (d) of TYM as key variable. Accordingly, we needed 25 persons in each group. Assuming 5 dropouts in each group, the final sample size was determined to be 30 persons per group. Figure 1 explains flow of subject selection assigned as CON and PRO groups enrolled in the study.

### Ethical approves

This clinical trial was done in accordance with the Helsinki Declaration of 1975. Also, performing the study was approved by the ethics committee of Kashan University of Medical Sciences (KUMS) and registered in the Iranian Website for Registration of Clinical Trials IRCT (IRCT number: 2017061534549N1). Written Informed consent was received from all patients.

### Study design

At the onset of the study, all subjects were matched for disease severity based on gender, BMI, and age. Participants were then randomly divided into two groups: the control (CON) group (*n* = 23 including 13 females and 10 males) receiving placebo capsules containing 500 mg maltodextrine and, the probiotic (PRO) group (*n* = 25: 18 females and 7 males) receiving capsules containing a mixture of probiotic bacteria (see below for details). The treatment was lasted for 12 weeks. Participants were requested to have no change in their regular physical activity and consume no nutritional supplements during the clinical trial.

### Intervention

It would be more appropriate to use strains of probiotics for human consumption derived from the human intestinal tract, well characterized, able to outlive the rigors of the digestive tract and possibly colonize, biologically active against the target as well as to be stable and amenable to commercial production and distribution (25). Very rare reports are evident about effectiveness of probiotic bacteria on cognitive phenomena (26–29). In our previous works we tested mixtures of probiotics containing both *Lactobacillus* and *Bifidobacterium* genera (*Lactobacillus acidophilus, Lactobacillus casei, Lactobacillus fermentum*, and *Bifidobacterium bifidum*). In the present study we tested the effect of other multispecies probiotics containing different stains and species of the genera *Lactobacillus* and *Bifidobacterium*. We prepared two types of capsules each containing 3 bacteria (with a total dosage of 3 × 10^9^ CFU) including either *Lactobacillus fermentum, Lactobacillus plantarum*, and *Bifidobacterium lactis* (provided by Zist Takhmir Company, Tehran, Iran) or *Lactobacillus acidophilus, Bifidobacterium bifidum*, and *Bifidobacterium longum* (provided by Milad Farmed Company, Tehran, Iran). The probiotic group received one of each capsule every other day.

### Anthropometric considerations

Weight and height of the participants were determined in an overnight fasting status using a standard scale (Seca, Hamburg, Germany) before and 12 weeks after the treatment. BMI was calculated as: weight (Kg)/height (m^2^).

### Outcome evaluation

The TYM test was used to evaluate the level of cognition in the patients and the TYM results were considered as the primary outcomes. However, the two questions in the semantic knowledge section of the TYM were substituted by questions familiar to Iranian people. In the first question the subjects were asked to name the Iranian president rather than the UK prime minister. Also the second question of semantic knowledge “In what year did the 1st World War start?” uncommon knowledge in Iran, was changed to “In what year did the war between Iran and Iraq?” As a secondary outcome the findings of the oxidative stress and inflammatory biomarkers were assessed.

### Assessment of biochemical parameters

Twelve-hour fasting blood samples were collected by venipuncture before and after the intervention. The blood samples were taken according to a standard protocol and centrifuged (Hettich D-78532, Tuttlingen, Germany). Then, the samples were stored at −80°C until analysis. Serum total antioxidant capacity (TAC) was quantified using the method of ferric reducing antioxidant power method developed by Benzie and Strain (30). The method of Beutler et al. was used for measuring total glutathione (GSH) (31). Plasma concentration of malondialdehyde (MDA) was measured by the thiobarbituric acid reactive substance method (32).

The serum high sensitivity concentrations of IL-6, IL-10, and TNF-α were measured using commercial ELISA kit (Diaclone, French). Serum high sensitivity 8-hydroxy-2′ -deoxyguanosine (8-OHdG) concentration was also quantified by use of commercial ELISA kit (Zelbio, Germany). Plasma nitric oxide (NO) was quantified by the Griess method (33).

### Statistical methods

Kolmogrov-Smirnov test was applied to the data to determine normal distribution of the variables. Possible differences in anthropometric measures were detected using unpaired student *t*-test. The effect of probiotic supplementation on the TYM cognitive test, oxidative stress biomarkers, and inflammatory factors, determined by one-way analysis of variance (ANOVA) followed by Tukey's post test. The change before and after the intervention was calculated as: % (post-treatment value–pre-treatment value)/ pre-treatment value. The differences between the healthy and AD participants, between the participants with moderate and severe AD, and between the changes in the CON and PRO groups were determined by unpaired student *t*-test. The data are reported as mean ± SEM. The differences were significant if *P* value was <0.05. All statistical analyses were performed using SPSS—version 18.

## Results

### Anthropometric characteristics of the patients

The patients assigned to the CON and PRO groups weighed 80.57 ± 1.79 and 79.70 ± 1.72 kg, respectively at the onset of the study. BMI index of the subjects enrolled in the study showed no difference over 12 weeks. The change percent of BMI was 0.11 ± 0.29 and 0.74 ± 0.34 in the CON and PRO groups (*P* > 0.05), respectively (Table 1).

**Table 1:** Anthropometric characteristics of the patients.

		**CON group (*n* = 23)**	**PRO group (*n* = 25)**	***P* value**
**Gender**	Male	10	7	0.42
	Female	13	18	0.25
Age (year)		80.57 ± 1.79	79.70 ± 1.72	0.36
**Weight (kg)**	Before treatment	60.63 ± 1.26	60.12 ± 1.12	0.42
	After treatment	60.58 ± 2.36	60.32 ± 1.42	0.48
Height (cm)		156.43 ± 1.86	156.77 ± 1.23	0.38
**BMI (kg/m**^2^**)**	Before treatment	24.44 ± 1.33	24.05 ± 1.07	0.47
	After treatment	24.56 ± 1.34	24.21 ± 1.06	0.45

*CON, control; PRO, Probiotic*.

### Confirmation of Alzheimer's disease in the patients based on the TYM cognitive test

**Figure 2:**
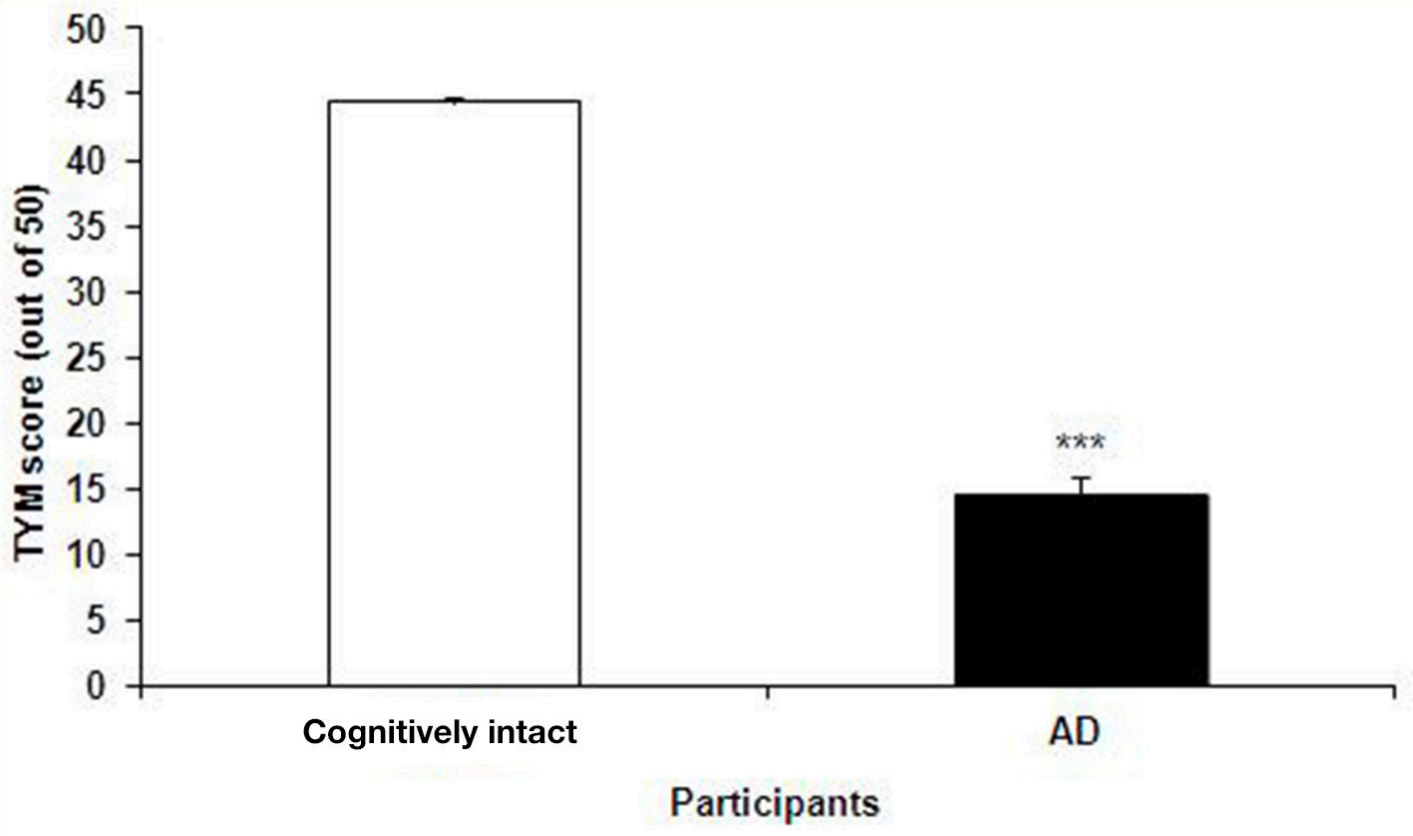
The scores of the TYM test achieved by the cognitively intact people and those with Alzheimer's disease (AD). The scores gained by the cognitively intact and AD participants were 44.55 ± 0.37 against 14.51 ± 1.40, respectively. Unpaired student *t*-test indicated a significant difference between the two groups (^***^P < 0.0001).

**Figure 3:**
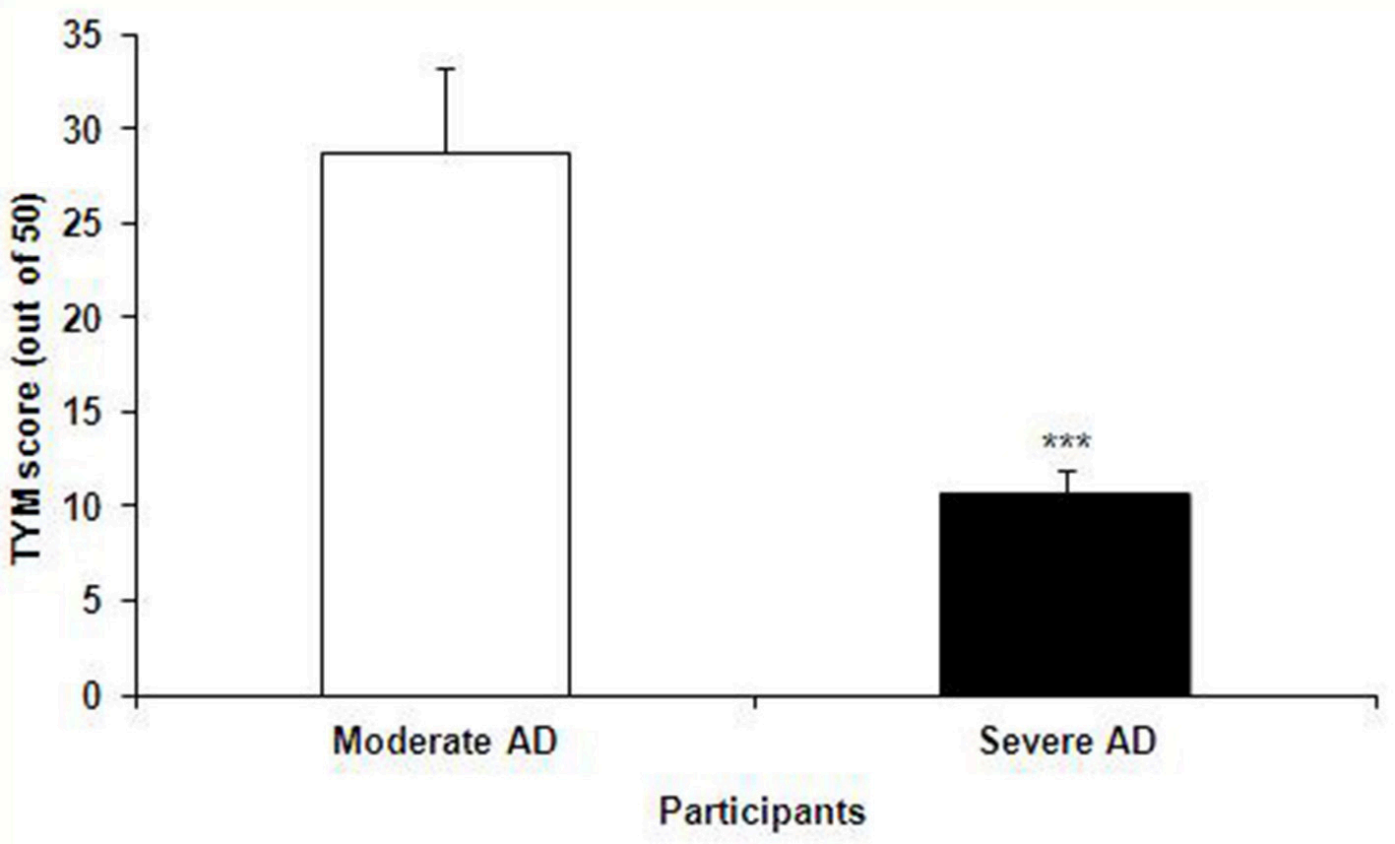
The TYM scores achieved by the patients with moderate and severe Alzheimer's disease (AD); the former group gained 28.7 ± 4.47 score and the latter 10.63 ± 1.35 score. Unpaired student *t*-test showed a significant difference between the two AD groups (***P < 0.0001).

The TYM test was used to further confirm the healthy and Alzheimer's subjects. TYM is a cognitive tool designed for detection of AD that includes 10 cognitive tasks with a total of 50 scores. Therefore, the participants were firstly introduced to the TYM cognitive test. The subjects assigned as cognitively intact people gained an average of 44.55 ± 0.37 scores. This value is near to the criterion in the TYM test (34) to confirm lack of any dementia. On the other hand, the people assigned as AD patients committed an average of 14.51 ± 1.40 scores. Such a score in the TYM test fall in range of severe AD (scores <25). Unpaired student *t*-test indicated a considerable significant (*P* < 0.0001) between the cognitively intact people and those with AD (Figure 2). Then, the AD patients were divided in to two CON and PRO group treated by placebo (maltodextrine) and probiotic bacteria, respectively. Of them, a majority of 83.5% showed a severe AD and the rest (16.5%) had a moderate disease. The people with severe AD gained 10.63 ± 1.35 score and those with moderate AD gained 28.7 ± 4.47 score. The scores achieved by the moderate and severe AD patients entered the study are illustrated in the Figure 3. In the CON patients 17.86% were fallen in a range of moderate AD committing a mean score of 30 ± 4.62. The rest (82.14%) of the patients fell in a range of severe AD, gaining a score of 9.57 ± 1.37. In the PRO group, 16.13% of patients gained 27.14 ± 4.32 scores showing a moderate AD. The rest of patients (*n* = 26, 83.87%) gained 11.69 ± 1.33 scores confirming a severe disease. Consequently, the percentage of patients with mild and severe disease was almost the same in both CON and PRO groups.

### The effect of probiotic supplementation on TYM test

The TYM cognitive test was taken from the CON and PRO group before and 12 weeks after the intervention. The pre- and post-treatment TYM scores in the PRO group were 14.64 ± 1.71 and 17.42 ± 2.42, respectively; the values in the CON patients were 14.35 ± 2.27 and 17.47 ± 2.89, respectively (Table 2). Analysis of variance indicated no difference between the two groups [*F*_(3, 114)_ = 0.29, *P* < 0.82]. The change percent between the scores achieved at the onset and offset of the trials was 12.86% ± 8.33 in the CON group and −9.35% ± 16.83 in the PRO group, however, the unpaired student *t*-test indicates no statistical difference between the two groups (Table 3).

**Table 2:** Pre- and post-treatment cognitive scores and biochemical values in the CON and PRO groups.

	**CON group**		**PRO group**		**Difference between the two groups**
	**Pre-treatment**	**Post-treatment**	**Pre-treatment**	**Post-treatment**	***P* value**
TYM (score out of 50)	14.35 ± 2.27	17.47 ± 2.89	14.64 ± 1.71	17.42 ± 2.42	0.82
TAC (μmol/L)	868.75 ± 42.45	885.72 ± 29.05	1029.97 ± 23.41	937.07 ± 16.06	< 0.01
GSH (μmol/L)	640.78 ± 19.77	731.55 ± 37.33	727.82 ± 22.01	759.01 ± 28.29	>0.05
MDA (μmol/L)	3.07 ± 0.07	2.98 ± 0.07	3.20 ± 0.07	2.87 ± 0.06	< 0.05
NO (μmol/L)	39.12 ± 1.81	47.75 ± 3.45	35.94 ± 2.43	38.06 ± 0.75	< 0.01
TNF-α(pg/ml)	0.63 ± 0.03	2.13 ± 1.07	1.89 ± 0.72	1.56 ± 0.44	>0.05
IL-10(pg/ml)	0.59 ± 0.01	0.76 ± 0.17	0.73 ± 0.12	0.59 ± 0.01	>0.05
IL-6(pg/ml)	3.58 ± 1.04	3.37 ± 1.41	3.42 ± 0.61	4.51 ± 1.29	>0.05
8-OHdG(ng/ml)	43.72 ± 6.31	41.80 ± 6.19	43.25 ± 3.01	42.70 ± 3.27	>0.05

*The values in the two groups are compared by one-way ANOVA followed by Tukey's post-test*.

*CON, control; PRO, Probiotic*.

**Table 3:** Comparison of the change percent of the biochemical factors between the control and probiotic groups.

	**CON Group (Change%)**	**PRO group (Change%)**	***P* value**
TYM (score out of 50)	0.12 ± 0.08	−9.35 ± 16.83	>0.05
TAC (μmol/L)	0.07 ± 0.07	−0.06 ± 0.03	0.058
GSH (μmol/L)	0.08 ± 0.05	0.04 ± 0.03	0.26
MDA (μmol/L)	−0.11 ± 0.03	−0.17 ± 0.03	0.11
NO (μmol/L)	0.11 ± 0.06	0.05 ± 0.09	0.30
TNF-α (pg/ml)	0.35 ± 0.17	−0.15 ± 0.27	>0.05
IL-10 (pg/ml)	0.05 ± 0.10	−0.70 ± 0.73	>0.05
IL-6 (pg/ml)	−1.67 ± 1.33	2.18 ± 0.15	<0.001
8-OHdG (ng/ml)	−0.33 ± 0.37	−0.02 ± 0.12	0.21

*The values between the two groups were compared by unpaired student t-test*.

*CON, control; PRO, Probiotic*.

### Inflammatory/anti-inflammatory factors

Analysis of variance applied on the data taken from the CON and PRO groups showed that treatment of the AD patients with the probiotic supplementation not significantly affected TNF-α [*F*_(3, 63)_ = 0.64; *P* > 0.05], IL-6 [*F*_(3, 71)_ = 0.27; *P* > 0.05], and IL-10 [*F*_(3, 63)_ = 0.64; *P* > 0.05]. The change percent in the serum level of TNF-α, IL-6, and IL-10 in the CON group was 0.35% ± 0.17, −1.67% ± 1.33, and 0.05% ± 0.10, respectively. The values in the PRO group were −0.15% ± 0.27, 2.18% ± 0.15, and −0.70% ± 0.73, respectively. The pre- and post-treatment values are shown in the Table 2 and the change percent are given in the Table 3.

### Oxidants/antioxidants factors

A general statistical difference was observed concerning TAC between the two group [*F*_(3, 81)_ = 4.42, *P* < 0.01]. However, within group analysis indicated no considerable difference between the pre- and post-treatment values in the two groups. The change percent in the CON and PRO groups were 0.07% ± 0.07 and −0.06% ± 0.03, respectively.

We observed no variation in the serum level of GSH in the two testing groups [*F*_(3, 82)_ = 4.25, *P* > 0.05]. The change percent in the CON and PRO groups were 0.08% ± 0.05 and 0.04% ± 0.03, respectively.

Although ANOVA showed a significant difference in serum level of NO between the CON and PRO patients [*F*_(3, 81)_ = 4.42, *P* < 0.01, Table 2], however, the *post-hoc* test indicates that the change percent in the CON (0.11% ± 0.06) and PRO (0.05% ± 0.09) groups was ignorable (Table 3). Despite ANOVA confirms a general variation in the MDA concentration of serum in the CON and PRO groups [*F*_(3, 82)_ = 3.50, *P* < 0.05, Table 2] the post-test indicates a negligible change percent of MDA in the CON (−0.11% ± 0.03) and PRO (−0.17% ± 0.03) patents (Table 3). The pre- and post-treatment change in the serum level of the DNA degrading factor 8-OHdG showed not statistically to be considerable [*F*_(3, 71)_ = 0.02, *P* > 0.05]. The change percent values are −0.33 ± 0.37 and −0.02 ± 0.12, respectively. The Table 2 explains analysis of pre- and post-treatment values in the CON and PRO AD patients based on ANOVA. The change percent in the CON and PRO groups analyzed based on unpaired student *t*-test are shown in Table 3.

## Discussion

The pathologic features of AD are multifaceted, including precipitation of extracellular amyloid-β plaques, formation of intracellular neurofibrillary tangles and damage to neuronal synapses (28). However, these aspects of AD are easily researchable in experimental studies on animal models. Therefore, in level of investigation, histological changes in the AD patients were not under focus of this study. Despite, some other indications which are also observable in AD patients are measurable in clinical researches. Hence, this study was devoted to evaluation of alterations in the oxidants/antioxidant and inflammatory/anti-inflammatory factors as well as cognitive status in the AD patients.

We asked if reinforcement of the gut microbiota by probiotic bacteria influences the cognitive as well as biochemical disorders in the AD patients. Using the TYM test, cognitive status of the AD patients was analyzed. As illustrated in the Figure 2 the scores achieved by the AD patients are less than one third compared to the cognitively intact people, confirming a real dementia in the group assigned as AD. The AD patients were given a formula of probiotic supplementation consisting both Lactobacilli and Bifidobacteria. The cognitive TYM assessment indicated no difference between the CON and PRO groups. Our results showed that the intervention not pronouncedly influenced either inflammatory (TNF-α and IL-6) or anti-inflammatory (IL-10) factors. Also, our findings indicated that the oral bacteriotherapy had no considerable effect on the oxidants (MDA and 8-OHdG) or antioxidant (TAC, GSH) factors.

In healthy humans, there is a close mutualistic and symbiotic relationship between gut microbiota and the body. This normal state of the human intestinal microbiota is called eubiosis. Any distortion from eubiosis, linked with whether a decrease of intestinal biodiversity or increase of pathogenic bacteria, is called dysbiosis. The dysbiosis results in alteration of the immune system of the gut mucosa and the rise of inflammatory, immune, metabolic or degenerative diseases (35). Emerging evidence indicates that change in gut microbiota composition may contribute in some neurological disorders. Therefore, intervenes like antibiotics, probiotics, pathogens, and nutrition are expected to affect the composition of gut microbiota and physiological function of gut and, thus, influence the host cognitive behavior and change the risk of AD (36). Particularly, nutritional interventions are now promising as strategies to treat or at least delay AD progression.

Very rare preclinical findings are evident on the effect of probiotic supplements on AD. Recently, Bonfili et al. reported that treating an animal model of AD (mice 3xTg-AD) with a probiotic formulation (SLAB51) positively interfere with inflammatory cytokines and concentration of some gut hormones, reduce amyloid-β plaques and improve cognitive function (26). In another study Nimgampalle and Kuna revealed that *Lactobacillus plantarum* MTCC1325 might have anti-Alzheimer properties against D-Galactose induced AD (27). Further support was provided by Peng et al. where they reported that the probiotic bacteria *Lactobacillus plantarum* NDC75017 improves the learning and memory ability in aging rats (37). Also Distrutti et al. demonstrated that a formulation of probiotic (VSL#3) positively affected inflammatory and neuronal processes and restored impaired synaptic plasticity (38). In a research on healthy humans Chung et al. reported that fermented milk containing *Lactobacillus helveticus* IDCC3801 improves cognitive function in healthy older adults (29).

We were the first to present a clinical work demonstrating a favorable effect of probiotic bacteria on the patients with AD. Three months treating the AD patients with a probiotic formulation containing four bacteria (*Lactobacillus acidophilus, Lactobacillus casei, Lactobacillus fermentum*, and *Bifidobacterium bifidum*) slightly improved cognitive indices, increased some antioxidant factors and normalized some lipid profiles (21). However, compared to the previous work, more AD patients were fallen in severe stage in the present study (83.5 vs. 67%) and less in moderate stage (16.5 vs. 33%). In another clinical trial we also found positive effect of a probiotic supplementation on motor behavior, and plasma level and gene expression of some biochemical indices in the people with multiple sclerosis (39, 40).

Lack of inflammatory biomarkers measurement in the AD patients was considered as a limitation in our previous work. Hence, in the present study the inflammatory cytokines TNF-α, IL-6, and IL-10 were evaluated. Increased TNF-α is known as a key element in inflammatory cascade that in turn increases the amyloid-β and tau pathology in AD (41). Evidence indicates an enhanced expression of IL-6 in both the periphery (42) and the CNS (43) in AD patients. Concerning IL-10, both positive (44) and negative (45) effects of this cytokine on cognition of AD animal models are reported. However, the probiotic supplement used in this study had no effect on either inflammatory or anti-inflammatory parameters in the AD patients. Some other inflammatory biomarkers related to the cognitive disorders must also be considered. Two of them are S100A12 and neopterin, known as intestinal and systemic inflammatory biomarkers, respectively. In a recent work Leblhuber et al. found a close correlation between fecal S100A12 and serum neopterin in cognitive disorders like AD; signifying a role of gut inflammation as a possible pathogenic cofactor in cognitive deterioration and dementia (34).

A key mechanism associated with AD is oxidative stress (46) that might be modified through nutritional or antioxidant supplements (47). Our findings indicated no effect of the probiotic supplementation on either oxidant or antioxidant biomarkers. In a clinical trial Tramutola et al. reported that AD patients were irresponsible to the administered antioxidants (48). They concluded that clinical trials of drugs with only a single mechanism against AD at the dementia stage might be expected.

Findings of this clinical trial indicated that treatment of the AD patients with a formulation of the probiotic bacteria effectively influenced neither the cognitive function nor biochemical factors. In our previous work on the AD patients we found a favorable effect of a different probiotic supplementation on both the cognition and some of biochemical statuses. We think that the difference between results of the two studies could be attributed to the different probiotic formulation and severity of the disease. However, some other reasons might explain why application of supplements on aged patients such as people with AD no sufficiently affects the cognitive and biochemical factors. A reason could be the protocol including the duration of the supplement administration. In a recent clinical trial conducted on more than 7,000 men with AD Kryscio, et al found that supplements containing vitamin E, selenium, or vitamin E + selenium had no effect on dementia (49). Also, wrong timing of administration may be responsible for the failure of supplements to influence the age associated disorders where, in sever stage of AD, the loss of synapses and development of neurofibrillary tangles are irreversible pathological changes (50). Concomitantly, the use of probiotic at this stage may not be successful in reversing the disease process. Eventually, it is worthwhile to imply that inflammatory/oxidative stress pathways are not specific to AD and are sensitive pathways involved in many diseases. Thus, we have tested the effect of probiotic supplementation on cytokines and oxidative stress pathways that might be only a part of the pathophysiology of the AD.

Conclusively, inconsistent effects of supplements on neurological disorders in aged people must not be interpreted as paradoxical results. Other intervenes including age and severity of disease must be considered in future studies.

There were some limitations that influenced our study; importantly inclusion of people mostly in severe stage of AD, small number of subjects included in the study, the dosage and formulation of probiotic bacteria, and a sort supplement exposure time. Additionally, assessment of the impaired cognitive related inflammatory biomarkers, S100A12, and neopterin, are suggested in future investigations on AD patients.

## Author contributions

MSa designed the project. AAg, AAl, MSo, and SE (general physician) performed the study. RD (neurologist) and MH (psychiatrist) visited the participants. GH analyzed the data. The manuscript was written by MSa. Final edit was accomplished by MSa.

### Conflict of interest statement

The authors declare that the research was conducted in the absence of any commercial or financial relationships that could be construed as a potential conflict of interest. The reviewer YT and handling Editor declared their shared affiliation.
