# Corrigendum: Does Severity of Alzheimer's Disease Contribute to Its Responsiveness to Modifying Gut Microbiota? A Double Blind Clinical Trial

**DOI:** 10.3389/fneur.2019.00031

**Published:** 2019-02-05

**Authors:** Azadeh Agahi, Gholam Ali Hamidi, Reza Daneshvar, Mostafa Hamdieh, Masoud Soheili, Azam Alinaghipour, Seyyed Mohammad Esmaeili Taba, Mahmoud Salami

**Affiliations:** ^1^Physiology Research Center, Kashan University of Medical Sciences, Kashan, Iran; ^2^Department of Neurology, School of Medicine, Kashan University of Medical Sciences, Kashan, Iran; ^3^Department of Psychology, School of Medicine, Shaheed Beheshti University of Medical Sciences, Tehran, Iran; ^4^Taleghani Branch, Department of Education, Farhangian University, Qom, Iran

**Keywords:** probiotics, Alzheimer's disease, cognition, inflammation, oxidative stress, microbiota

In the original article, there was an error. The term “normal people” was used instead of the term “cognitively intact people.”

Corrections of this term have been made throughout the text in the **Materials and Methods, participants**; the **Results**, **Confirmation of Alzheimer's Disease in the Patients Based on the TYM Cognitive Test**; Figure 2 and in the **Discussion**, paragraph one.

Additionally, the determination of the sample size was incorrect. A correction has been made to the **Materials and Methods**, **Participants:**

“This clinical study was performed as a randomized, double-blind, and placebo-controlled clinical trial. Participants included in this study were people with AD (65–90 years old) residing at Emam Ali (Tehran, Iran), Golabchi (Kashan, Iran), Miad (Kashan, Ravand, Iran), and Barekat (Aran and Bidgol, Iran) Welfare Organizations between June 2017 and August 2017. AD patients were diagnosed based on the NINDS-ADRDA criteria (22) and revised criteria from the National Institute on Aging-Alzheimer's Association (23). For further proof the AD patients were compared with the cognitively intact people based on the TYM cognitive test. Accordingly, the participants gaining TYM scores in level of AD (<45 out of 50 scores) were entered the study (see section “Results” for details). Patients with metabolic disorders, chronic infections and/or other clinically relevant disorders were excluded from the study. Standard formula for clinical trials was used to calculate sample size for the study. Based on a previous study (24), considering type one error (α) of 0.05 and type two error (β) of 0.20 (power = 80%) we used 1.3 as SD and 1.1 as the difference in mean (d) of TYM as key variable. Accordingly, we needed 25 persons in each group. Assuming 5 dropouts in each group, the final sample size was determined to be 30 persons per group. Figure 1 explains flow of subject selection assigned as CON and PRO groups enrolled in the study.” Furthermore, there was an error in the use of the term “underpowered.” A correction has been made to the **Discussion**, Paragraph 10:

“There were some limitations that influenced our study; importantly inclusion of people mostly in severe stage of AD, small number of subjects included in the study, the dosage and formulation of probiotic bacteria, and a sort supplement exposure time. Additionally, assessment of the impaired cognitive related inflammatory biomarkers, S100A12, and neopterin, are suggested in future investigations on AD patients.”

Lastly, there was a missing translation of the TYM test. A correction has been made to the **Material and Methods**, **Outcome Evaluation**:

“The TYM test was used to evaluate the level of cognition in the patients and the TYM results were considered as the primary outcomes. However, the two questions in the semantic knowledge section of the TYM were substituted by questions familiar to Iranian people. In the first question the subjects were asked to name the Iranian president rather than the UK prime minister. Also the second question of semantic knowledge “In what year did the 1st World War start?” uncommon knowledge in Iran, was changed to “In what year did the war between Iran and Iraq?” As a secondary outcome the findings of the oxidative stress and inflammatory biomarkers were assessed.”

The authors apologize for these errors and state that this does not change the scientific conclusions of the article in any way. The original article has been updated.

